# Alcohol and fatal life trajectories in Russia: understanding narrative accounts of premature male death in the family

**DOI:** 10.1186/1471-2458-11-481

**Published:** 2011-06-20

**Authors:** Lyudmila Saburova, Katherine Keenan, Natalia Bobrova, David A Leon, Diana Elbourne

**Affiliations:** 1 Department of Sociology, Izhevsk State Technical University, 7 Studencheskaya Street, Social, Izhevsk, 426069, Russia; 2 Faculty of Epidemiology and Population Health, London School of Hygiene and Tropical Medicine, Keppel Street, London, WC1E 7HT, UK; 3 Faculty of Epidemiology & Public Health, University College London, 1 -19 Torrington Place, London, WC1E 7HB, UK

## Abstract

**Background:**

In the post-Soviet period, Russian working-age men have suffered unusually high mortality rates. Earlier quantitative work found that part of this is attributable to hazardous and harmful patterns of alcohol consumption, which increased in the period of transition at a time of massive social and economic disruption and uncertainty. However, there has been very little work done to document and understand in detail the downward life trajectories of individual men who died prematurely from alcohol-related conditions. Building on an earlier case-control study, this unique qualitative study investigates the perceived interplay between men's drinking careers, their employment and family history, health and eventual death.

**Methods:**

In-depth interviews were conducted with close relatives (most often the widow) of 19 men who died between 2003 and 2005 aged 25-54 years whose close relatives reported that alcohol contributed to their death. The study was conducted in a typical medium-sized Russian city. The relative's accounts were analysed using thematic content analysis.

**Results:**

The accounts describe how hazardous drinking both contributed to serious employment, family and health problems, and was simultaneously used as a coping mechanism to deal with life crises and a decline in social status. The interviews highlighted the importance of the workplace and employment status for shaping men's drinking patterns. Common themes emerged around a culture of drinking in the workplace, peer pressure from colleagues to drink, use of alcohol as remuneration, consuming non-beverage alcohols, Russian-specific drinking patterns, attitudes to treatment, and passive attitudes towards health and drinking.

**Conclusions:**

The study provides a unique insight into the personal decline that lies behind the extremely high working-age mortality due to heavy drinking in Russia, and highlights how health status and hazardous drinking are often closely intertwined with economic and social functioning. Descriptions of the development of drinking careers, hazardous drinking patterns and treatment experiences can be used to plan effective interventions relevant in the Russian context.

## Background

Since the fall of the Soviet Union, mortality rates of Russian men have experienced dramatic fluctuations, diverging considerably from patterns seen in other industrialised countries. Male life expectancy decreased to a low of 57 years in 1994, and despite a modest increase in recent times to approximately 62 years, still lags considerably behind other European nations [1,2].The male mortality crisis is driven by high death rates among working-age men, and there is strong evidence that alcohol plays an important role in these trends [1,3-9].

Alongside high overall consumption [10] the Russian drinking pattern includes particularly hazardous features, such as *zapoi*. This is a well recognised term in Russia to describe an extended episode of heavy drinking characterised by a period of continuous drunkenness lasting at least two days, where the person withdraws from normal life[6]. A recent survey found that 10% of working-age Russian men reported going on *zapoi *in the last year [11]. This phenomenon should not be confused with what is commonly defined as binge drinking in many Western countries. For example, in 2004 the US National Institute on Alcohol Abuse and Alcoholism (NIAAA) defined binge drinking as "a pattern of drinking alcohol that brings blood alcohol concentration (BAC) to 0.08 gram percent or above. For the typical adult, this pattern corresponds to consuming 5 or more drinks (male), or 4 or more drinks (female), in about 2 hours"[12]. While almost all episodes of *zapoi *would meet this definition of a binge, very few episodes of binge drinking in Western countries would be classed as *zapoi*.

In addition, in both clinical and general populations, consumption of non-beverage alcohols was reported as relatively common [11,13]. These are manufactured, legal alcohols not intended for drinking, such as medicinal tinctures and eau du colognes which in this paper are referred to as surrogates [11]. Surrogates typically contain 60-95% alcohol content, and in terms of pure ethanol, are considerably cheaper than vodka [14,15]. This qualitative research builds on a previous case-control study showing that working-age men who experienced either of these behaviours- *zapoi *or consumption of surrogates - had a significantly higher risk of premature death [6].

In Russia the treatment of alcohol problems is highly medicalised[16], and mainly delivered through specialist institutions (narcology dispensaries). While the available treatments include ones described as psychotherapeutic, they are highly directive. They include a procedure known as "coding" (*kodirovanie)*. Raikhel [17] describes how coding works by persuading the patient that the narcologist has altered their brain so that consumption of alcohol is physically dangerous. It became popular in the 1980s and 90s in a highly commercialised form and was marketed as a 'magic bullet' cure. Like placebo therapy (*khimzashchita*) it relies on techniques of suggestion and the perceived expertise and authority of the narcologist. While coding is regarded as a means of inducing a placebo effect, active pharmacological interventions such as disulfiram that produce unpleasant reactions if the person drinks are also employed [17]. The aim of most treatments in Russia is to achieve a "cure" or complete abstention, rather than harm reduction[18]. There has been little use of person-centred individual counselling. Treatment at narcology dispensaries or psychiatric hospitals is usually without charge to the patient, many being admitted either because of an acute medical or psychiatric episode induced by alcohol or they are required to undergo treatment by the courts as a result of having been charged with an offence. In some parts of Russia there is a relatively developed private sector for treatment of alcohol problems, although this will only be affordable to a minority of the population.

Increases in male hazardous drinking and mortality occurred in the 1990s, during a time of widespread social and economic uncertainty, resulting from the sudden collapse and disappearance of the Soviet Union [19]. During the early 1990s, economic 'shock therapy' led to the population suffering a drastic fall in living standards, rising rates of unemployment, and salary delays[20]. This was in part driven by a rapid and chaotic programme of privatisation of many parts of the economy, which in turn has been linked to the increases in mortality seen in Russia and other Eastern European countries during the transition [21]. Economic inequality increased rapidly and hardships were exacerbated by the sudden collapse of the Soviet social safety net [22,23].

It has been argued that men in Russia were particularly vulnerable to changes in work and social life that occurred in the early transition period. This is because Russians hold neo-traditional conceptions of family life and masculinity, and perceive a strict division of the domestic and wage-earning spheres [24,25]. Men who become unemployed lose their core social and familial identity of breadwinner and may be more likely to drink to compensate for the loss of status [25]. These arguments are supported by data showing that men with lower education, who are unmarried, and unemployed, are more likely to be problem drinkers, and have higher mortality rates [11,26]. However, as most studies use cross-sectional data, evidence for a causal relationship between economic, social factors and heavy drinking in men is limited.

The vast majority of the research investigating drinking and mortality in Russia uses quantitative methods. There is a dearth of qualitative studies, with some notable exceptions [13,27-29]. Previous studies have tended to collect generalised or 'lay' perceptions. Our study uniquely illuminates how lived experience of premature death affects opinions about reasons for poor male health, men's heavy drinking, and gendered health behaviours. In addition, the majority of our respondents were widows of deceased men, giving the opportunity to explore how male heavy drinking in Russia is negotiated among couples, and directly affects family relationships.

In this paper we report the results of a qualitative study that uniquely focuses on men who died prematurely in the city of Izhevsk between 2003-05 through the accounts provided by family members who they lived with. The aims of the investigation were to throw light on the perceived pathways to death, and to understand how relatives understood the interplay between the men's drinking, employment, family and health history.

## Methods

The qualitative study was carried out in Izhevsk, the capital of the Udmurt Republic, on the western side of the Ural Mountains, approximately 1000 km east of Moscow. It is a medium sized industrial city, with a population of about 650,000 (2002 census) with a typical demographic profile. In 2009 approximately 30% of the workforce were employed in heavy industries such as engineering, metal working, and arms manufacture [30].

The aims were:

1. To identify common life-course trajectories of deceased men who had drunk heavily before death based on biographical narratives provided by relatives

2. To understand the relative's perception of attribution of ill health and death

3. To understand attitudes to problem drinking and alcohol treatment

The design and aims are similar to a verbal autopsy study, where one seeks to understand the perceived social aetiology of the disease, by generating an account of the life trajectory leading up to the death [31]. The subjectivity of the narratives does not threaten the reliability of the data, but is independently valuable, revealing attitudes towards drinking and premature death. Explanations for poor health and death may differ from 'lay' explanations found elsewhere [27] as the respondent has the concrete experience of living with a problem drinker, experiencing their poor health and death at close hand. Understanding the typical relationships between social factors, drinking and mortality can be used to generate new hypotheses for further research into causal mechanisms.

The research built on the Izhevsk Family study, a population-based case-control study, conducted between 2003-2005 to investigate the causes of working age male mortality, details of which are described elsewhere [6]. The case-control study used proxy informants (usually wife or partner) to find out about the circumstances and behaviours of the deceased men. The results showed that the deceased men had higher rates of problem drinking, lower education, unemployment, and divorce than the population-based live controls [6,26]. The information used in the case-control study was largely collected through fully-structured questionnaire interviews. While a considerable amount of information was collected on social and behavioural characteristics of the subjects, this could not be used to develop in-depth and informative accounts of the life trajectories of each man. Thus, to illuminate the quantitative findings, the qualitative study used semi-structured interviews with close relatives of the deceased man, where the interviewer elicited a biographical account of the life of the man covering his employment, drinking and family relations.

In the case-control study, proxy-respondents were interviewed for 1750 of the dead (case) men, 17% of whom declined to take part in further study. The remainder constituted the sampling frame for this qualitative study. A combination of purposive and stratified sampling was used. Purposive sampling was used to select only those proxies who stated that alcohol had contributed to the man's death, narrowing the potential cases to 582. Stratified sampling was then used to ensure a variety of drinking behaviours would be discussed at interview. The 582 cases were divided into strata according to incidence of Russia-specific drinking behaviours that were shown to have an adverse impact on health in the previous case-control study [6]. The 4 strata were: whether they drank surrogates in the year before death, went on *zapoi *in the last year, neither or both. The number of interviews was limited as the study was initially seen as a exploratory hypothesis-generating work for future longitudinal data collection. For practical reasons, therefore, four or five proxies were selected from each of the four stratum sequentially according to the date they were interviewed in the case-control study. This systematic sampling method is unlikely to introduce selection bias as the original date of interviews of the proxies was principally determined by the date of death of the man. In total we approached proxy informants of 19 dead men. All those selected were contacted by telephone, or in person, and all agreed to take part.

Oral consent was given to each interviewer before the interview commenced. This was consistent with the exclusive use of verbal consent in the case-control study itself. This approach had been approved by the ethical committees of the Izhevsk Medical Academy and the London School of Hygiene & Tropical Medicine, on the basis that in Russia there remains a substantial reluctance to sign any official document for fear of it leading to the individual being subsequently held to account by the authorities for what they said.

The 19 deceased men studied had a mean age at death of 45 years (range 28-54). As can be seen from Table 1 the majority had achieved specialised secondary education (technical college) and only 1 had higher education. More than half of the men were unemployed at the time of death, but the qualitative interviews showed that all had been employed in semi-skilled manual jobs for most of their lives; typically as electricians, plumbers, fitters or motor mechanics. Eighteen of the 19 cases had been married or divorced, and nearly all had children. The official causes of death of the men were collected as part of the original case-control study, and were coded by the certifying doctor or pathologist who performed the autopsy, using the tenth revision of the International Classification of Diseases. The causes of death consisted of 6 deaths from heart disease (of which 3 alcohol-related), 2 from liver disease, 3 from pneumonia, 1 from tuberculosis, and 7 from external causes. The external causes consisted of 3 suicides, 1 alcohol poisoning, and 2 other accidental poisonings. For 14 of the 19 deceased men the proxy was the wife or ex-wife of the deceased. The proxies for the other 5 cases were siblings, an adult son, a mother, and a cousin.

**Table 1:** Socio-economic characteristics of the deceased men, whose relatives were interviewed in the study

Characteristic at the time of death	Deceased men (N = 19)
Education N (%)	
Incomplete secondary or lower	3 (16)
Secondary, specialised and professional	15 (79)
Higher	1 (5)

Employment status N (%)	
Regular paid employment	8 (42)
Unemployed	10 (53)
Retired (due to age or invalidity)	1 (5)

Marital Status N (%)	
Married/Cohabiting	13 (68)
Divorced/Separated	5 (26)
Widower	0
Never Married	1 (5)

Most of the 19 interviews were one-on-one, but in a few cases one or more household members were present. They took place between January-May 2005, lasted anywhere from 20 to 60 minutes, and the majority took place in the homes of the interviewees. One took place at the subject's sister's workplace. Each interview was conducted by one of three female Russian interviewers (including one of the authors, LS). All had considerable experience in undertaking both quantitative and qualitative investigations. A topic guide developed for the study was used by all interviewers which had question prompts about the man's drinking career, employment history, family background and relations, and illness history.

When possible the interviews were recorded and transcribed. In all cases, the interviewer took detailed contemporaneous notes and wrote up a structured written interview report immediately after, which followed the same structure of the interview, but did not necessarily include verbatim responses. The material was professionally translated into English, and the unique subject numbers used in the earlier study replaced by numbers 1-19 (shown as #1-19 in the text) to protect anonymity. Initial coding was done in Russian by the first author (LS); secondary coding using translated transcripts was carried out by KK. Coding schemes were shared and any disagreements discussed and resolved, paying close attention to the cultural context. The transcripts were analysed using thematic content analysis, where the accounts were deconstructed and categorised into key themes which arose partly from the questions asked, and partly from the common themes in the interviewee's answers. The material was analysed in NVivo 8 using a 'cut and paste' method. From these common themes, typical life trajectory categories were developed. Quotes were chosen to support descriptions of key themes in the data, and illustrate deviant cases.

## Results

### Drinking Careers

The vast majority of the deceased were reported as drinking very heavily or continuously in the period leading up to their death. The early part of the man's drinking career, usually in his early adult life, was always described as self-controlled, functional drinking, where he drank only on social occasions, holidays, or with work colleagues ('good guys') when the drinking was accompanied by other social activities. This is contrasted with the descriptions of problem drinking, which was often indicated by moving from drinking beer and wine to drinking spirits or surrogates with other heavy drinkers, sometimes on a daily basis, drinking in the morning to treat a hangover, borrowing money for alcohol, frequent or longer *zapois*, and inability to stop drinking once he had started. Negative social consequences such as missing work, difficulty getting up, and not coming home because of *zapoi *were also frequently cited as indicators of a drink problem. The majority described how the man's heavy drinking and the first *zapois *started with work colleagues.

Fourteen of the nineteen men had drunk surrogates in the past; the most commonly mentioned brands were 'Hawthorn' a medicinal tincture (more than 70% alcohol) and 'Yason', marketed as 'perfumed water' (more than 80% alcohol), both of which were commonly available in pharmacies, and retail hardware shops in Izhevsk at the time of the interviews [15]. The use of surrogates has a long history in Russia, having been reported since pre-revolutionary times [32]. During the Soviet period, there many reports of factory workers drinking industrial alcohol, and theft of non-beverage ethanol by medical and laboratory staff, metal workers and engineering workers [32,33]. The practice becomes more prevalent when beverage alcohol is in short supply, such as in wartime, and during the Gorbachev anti-alcohol campaign in 1985-87. In this period there was a massive increase in sales of ethanol-based aftershaves, eau du colognes, and cleaning fluids, and reports of military personnel stealing and consuming alcohol from aircraft and tanks [32]. Among heavy drinkers, use of industrial alcohol and surrogates is commonplace[13].

In our study, without exception, the respondents said the deceased men drank surrogates not out of preference, but because they could not afford beverage alcohols.

"*When he stopped working he would drink Hawthorn because he didn't have money to buy vodka (it's cheap, it costs 10 roubles). Somebody recommended it to him."(#16)*

"He stopped working, had no money, drank Hawthorn, Yason, other bad staff every day.(#3)

The economic motivation fits with findings from previous studies [13]. In only very few accounts did a lack of money lead to the men abstaining or reducing their alcohol intake. Most respondents saw drinking surrogates as a sign the man had developed strong alcohol dependence, but there were a few cases where it was explained more as economic necessity than abuse. Most of the relatives believed that surrogates were unhealthy, (commonly described as "*bad stuff", "it can burn everything inside"*) but in some cases the risks could be mitigated by diluting them with water, which also was a signal that the man had some control over his drinking. Several relatives mentioned that the men refused to believe that surrogates carried health risks, even though they had been warned and nagged by their spouses and friends. Two of the men had severe adverse skin reactions to a particular brand of surrogate ("*he got covered with sores and ulcers" (#7)*, causing them to cease or to switch brands.

Longer and more frequent *zapois *were an indication of loss of control, increasing problem drinking and were accompanied by a gradual decline in health status. Many of the men went on *zapoi *around the time of their death and they lasted anywhere from 3 days to 2 months. *Zapoi *was described by the interviewees as uncontrollable drinking for long stretches, where the man misses work, doesn't eat, and enters a state where he loses power to stop drinking. In some instances a severe episode was only ended as a result of specialist intervention:

"*He got treatment only when I called for an ambulance for the first time. He couldn't stop zapoi. He had not eaten for ten days, had been unable to eat - everything had been in the basin [he vomited up what he had eaten]. He could not stand up. His legs did not support him. It was the first time I called for an ambulance. They said they could stop zapoi if we paid and took him to the narcology dispensary [Russian treatment centre for alcohol and drug problems]." (#2)*

It was common for the subject not to acknowledge their problem with alcohol and therefore refuse to engage in treatment. Some of the interviewees also denied the fact that the deceased were alcoholics, evidenced by of a lack of *zapoi*:

"*More recently he drank continuously but I don't think these were zapois. They say, zapoi is when a person even gets up at night to have a drink. He didn't drink at night and he never missed his work." (#16)*

"*Besides, can we say he was an alcoholic? He didn't have zapois. He got up in the mornings to help me; he went to collect bottles or something... Drank like everyone." (#1)*

It is interesting to note the subjective and contested definition of the term *'zapoi'*, which was understood slightly differently by each respondent, although it is generally understood to be a period of continuous drunkenness that by its very nature disrupts normal functioning.

Six of the men sought treatment enabling them to have periods of sobriety and return to normal life and work. Some of the men were unable to recognise their problem; those men that did felt helpless to deal with their dependence; some relatives mentioned that they felt disappointed by the treatment available. In Izhevsk, options for state-funded care is limited to narcology dispensaries or psychiatric departments treating severe cases of alcohol dependence, offering interventions to stop *zapoi*, detoxification programs, and 'coding' (see Background) to prevent consumption for a period of time. No psychological services are offered for free. Private clinics and doctors offer a broader range of services, including counselling, but the cost can be prohibitive.

### Drinking and Employment

In all cases, employment and drinking history were described as closely intertwined. In general, there were two, almost contradictory, reasons given for starting a heavy drinking career. In the first group, nine of the respondents felt that disruption in the man's workplace and loss of status directly or indirectly triggered heavy drinking. These men were often described as being happy, secure and successful early in their careers, before a 'crisis' situation at work such as redundancy, irregular pay, or conflicts with new management led them to drink more to deal with unhappiness or stress. The loss of stable employment status was identified as traumatic, leading directly to work dissatisfaction, loss of motivation and self-esteem, providing an almost logical reason for starting to drink heavily:

"In 1991 [the year of the collapse of the Soviet Union] everything changed. The plant was in a difficult financial situation. They didn't pay less but they had delays in wages. And very often the wages were not paid with money but with the plant's production. He couldn't adapt to such life. He tried to earn money, did two shifts in a row but at the same plant. He worked overtime. It was very difficult. He said he'd lived two lives already. That was the feeling he had. Gradually he drank more and more. Then he began drinking vodka. More recently he drank anything he could get" (#17)

Others suffered personal crises causing them to leave work and start drinking:

"*After another such conflict with her mother the girl [the subject's girlfriend] jumped from the window at the fifth floor. He suffered from deep depression. He said: "I don't want to live any more" and quit his second job. After that he didn't work for a year and a half, made friends with alcoholics who drank surrogates."(#9)*

On the other hand, in the second group, the remainder of the accounts described a gradual shift into heavy drinking, because they worked in places where alcohol was tolerated and encouraged and where they were pressured to drink with colleagues. Sometimes the men 'settled' for these types of jobs because they fitted in with their drinking habits. Certain professions, such as car mechanic, plumber and carpenter commonly received payment and tips in the form of alcohol rather than money. In these accounts, relatives often blamed the lack of regulation and social pressure in the workplace for the development of alcohol problems:

"*They alcoholise you in the [transport] fleet, I told him. How can I refuse, they ask me to do something with their car, he said. And of course, they give him a bottle, not money for this." (#14)*

"*He went to work and drank..*..*The management didn't pay attention (turned a blind eye) to the fact that the workers drink all the way through the shift. Nobody told them off. They set the machine working for the whole day and just periodically check that it's still going. And so [because of this]-you could do, what you wanted. Once he didn't get back home for three days. I found him in the shop. At nights he had slept there, drunk" (#11)*

*"It all started when he began working there, in that agency..*. *Before that he drank a little sometimes but never drank heavily" (#1)*

After heavy drinking developed, all describe patterns of subsequent unstable employment and lack of motivation to work, which lasted until death. Periods of employment were broken up with periods of heavy drinking, and ill health, and sometimes these factors were the reason for loss of employment:

"*But a little time after he fell off the wagon [went back to drinking heavily] - perhaps, something happened at work: he began drinking, missed work and was asked to leave... Then he got a job at the radio plant. They sacked him for drinking" (#15)*

"*He didn't work there long. Later he felt worse and worse. Would work a little, get back home and lie down. A few times I told him to go to the doctor. But he answered he wouldn't go anywhere. More recently he was registered at the job centre" (#13)*

The accounts describe how cycles of unemployment, drinking and ill health were mutually reinforcing, steadily bringing a decline in physical and mental health, making death seem inevitable. Rapid organisational changes in the workplace in the post-soviet period left some men unable to adapt, experiencing redundancy or salary delays, leading some of them to drink heavily, (see quotes above) while on the other hand, many were fired from their jobs because of alcohol abuse. Unemployment brought poverty, inducing many to drink surrogate alcohol as a cheaper alternative to beverage alcohol ("*He didn't have money to buy more vodka that's why he bought Hawthorn" (#13)*. Furthermore, it was perceived that drinking surrogates subsequently affected men's health, and brought about alcohol dependence, making them less likely to find a job, or more likely to be fired from the next job.

### Family and social life

In the majority of cases, men's heavy drinking, frequent *zapoi*, and surrogate use led to marital conflict, but not divorce. All but one of the deceased (the youngest who died at 28 years) had been married at some time and most had children. 12 were still married at the time of their death, and the other 6 were divorced or separated, 4 reportedly due to the husband's drinking. It was common for divorced couples to continue living together due to housing pressures.

Widows married at death tended to report good relations of mutual love, support and understanding ("*He confided in me" (#16) "He understood me. I was able to get from him what I wanted."(#2))*, mentioning that he was a good man despite his problems, and were more prone to see the deceased as a victim of circumstance. On the contrary, the divorced men were often blamed for their alcohol problem and subsequent death, ("*laziness was the main cause"(#8))*, recalled as being a burden to their families, and family relations were reported as non-existent or conflict-ridden.

Widows reported marital conflicts related to drinking but at the same time remained supportive, tolerant, and sympathetic, caring for the man until his final days. Five of the biographies reported inter-personal violence triggered by drunkenness:

*"He was very quiet, silent, a phlegmatic. But when he drank he became crazy, like a demon or a devil. And you know, I was afraid of him. He was 186 cm tall and weighed 120 kilos. The child used to restrain him, would get hold of him - don't touch mother*." *(#10)*

"We treated him the way he treated us. He would run after us with an axe, beat us badly, shout." (Son of deceased (#6))

Seven of the 19 interviews reported that the men had parents or siblings who were heavy drinkers or who went on *zapoi*, how this may have influenced the man was not discussed. Two of the widows mentioned that they drank together with their husbands.

In the accounts very heavy drinkers, especially surrogate drinkers, were stigmatised and became socially disconnected, either drinking in isolation, or spending time only with problem drinkers. The man's 'real' friends, perhaps from childhood, tended to drift away and to be replaced with a circle of drinkers. Drinking companions, although supportive and loyal in their own way, were a bad influence, encouraging him to drink surrogates, drink more regularly, and to borrow money.

### Attitudes towards Drinking, Illness & Death

The most common approach towards excessive drinking was for the spouse/relative to try to control his consumption, by persuading him to drink at home, rather than with colleagues or in the street, refusing to give money for alcohol, pouring away alcohol, and setting limits to the man's drunkenness in exchange for money, although these strategies had little effect.

On the other hand, many of the widow's accounts describe what might be labelled in the addiction field as 'co-dependent' and 'enabling' behaviour and attitudes,[34] comprising of excessive care-giving, and directly or indirectly helping the deceased to continue drinking heavily. For example, some of the women avoiding discussions or confrontations over alcohol:

*"We never had arguments - I always tried to avoid them....Never criticized him for drinking. Always tried to support him. When he was sober he was a very good man" (#15)*,

And some provided 'safe' home environments for drinking:

"The only thing I taught him not to do was not to drink in the street. You'd better come and drink at home to avoid problems, I told him. (#16)

Obvious examples of enabling, facilitative behaviour are widows providing money for strong alcohol and surrogates (9 of the proxies), drinking together with him "*so that less remained for him". (#1) *(2 cases), protecting men from the consequences of their drinking by providing excuses at their workplace (2 cases), and helping them to find another job after they had been sacked for drinking (3 cases). Relatives tended to be sympathetic and supportive if they were also concordant drinkers, or if they felt that alcohol was alleviating suffering:

"*When he was unable to walk any longer we bought it for him because he needed it. Otherwise he would cry without stopping. We diluted it well (a bottle for 1 or 1.5 litre) and left it next to his bed. It was enough for a day. Sometimes he forgot he'd already drank it. He'd drink and ask for it again. When he drank it he calmed down and had a sleep. More recently he would forget everything. He didn't eat anything during the last week. He only drank. He grew yellow." (#15)*

A few acknowledged that excessive tolerance on behalf of women could make them partly responsible for the problem:

"*Perhaps, we, the women, make them like this, we try to solve their problems, we nurse them" (#15)*

"I think to some extent mum is to blame for it too...when he's got a hangover mum carries fried eggs with gherkins to him. "Why, Mishen'ka [pet name for Mikhail] is suffering"(#15)

Most thought that treatment was pointless if the man didn't admit he had a problem, and a few rejected the idea of treatment completely, especially coding ("*I didn't insist on coding because I think coding is the end of the line*.(#7)"), describing alcoholism as an illness caused by innate weakness or a product of social forces.

For the suicides and sudden deaths particularly, it was common for the relative to have feelings of guilt, mentioning how they failed to prevent the fatality. Other relatives attributed blame to authorities for failing to diagnose and treat chronic diseases or adequately protect the man from harm. Where the illness was more protracted and death seemed inevitable, relatives tended to focus more on how he made his situation worse by avoiding medical intervention. However the genesis of illness was in most cases due to external causes, there was little discussion of the possibility of individual techniques for prevention. Only where the men died of alcohol poisoning was alcohol mentioned as a significant problem for physical health. Instead it was mainly mentioned that it impaired his social and economic functioning, or prevented him for seeking help for more serious medical conditions. It was common for interviewees to report that alcohol exacerbated long-standing chronic health problems that predated the problem drinking. Alcohol was sometimes a medication for pain and ailments.

Understandably, awareness of the issue of male problem drinking was high. Several of the respondents noticed increasing rates of drunkenness in particular neighbourhoods, and knew of other premature deaths related to drinking:

"Five people died from alcohol within the last three years. He was afraid but he thought it wouldn't happen to him" (#2)

"Another also died young. I don't know if it was related to drinking or not. We know it's poison but what can we do" (#1)

Some felt that heavier drinking was a result of post-Soviet changes: previously the Soviet state had provided more structured activities for schoolchildren and young people, job and wage security for adults which prevented problem drinking in men and young people. Young people and men were described as responding passively to alcohol advertising and difficult structural conditions, implying that state intervention and education were needed to solve the problem. Men were described as innately weaker than women, unable to resist invitations to drink, suffering from social stress more readily, lacking in ambition and passive in the face of health problems. By contrast, women's domestic responsibilities gave them strength and purpose

## Discussion

The deceased were commonly described as being caught in a downward spiral of heavy drinking, ill health, work and personal problems, where life problems were a motivation for heavier drinking, and heavier drinking, in turn, worsened the man's situation. In many cases, this vicious cycle meant that early death was perceived as inevitable. The mutual reinforcement between life crises and heavy drinking makes it difficult to determine generalisable causal relationships. Instead, this paper focuses on in-depth, detailed accounts to illuminate and contextualise the poor mortality statistics found in post-soviet Russia.

The employment context was understood to play a crucial role in the formation of men's drinking habits, with both drinking leading to unemployment, and work problems leading to heavy drinking. The men's biographies confirm that the relationship between heavy drinking and economic/social problems frequently runs in both directions, and in most cases it is difficult to separate them. Unstable employment was the most commonly cited explanation for starting a heavy drinking career. This could reflect the widely held idea that profession and stable employment is crucial for a man's sense of identity and self-esteem, and instability in this arena is understandable cause for drinking [22,27].

However at the same time most of the men's heavy drinking and *zapois *began in the workplace or with male colleagues, supporting previous research which suggested that the workplace is a very common, acceptable drinking context in Russia, much more so than in other countries [35]. Some industries were known for their drinking cultures, where even remuneration was sometimes in the form of alcohol, men are under peer pressure from colleagues to drink, and drinking was tolerated or encouraged by supervisors. The relationship between drinking and the workplace may reflect the fact that male socialising (which typically revolves around alcohol) [36] is relatively limited and strongly related to their workplace and work colleagues. As these accounts refer to the pre-Soviet or immediate post-Soviet workplaces, further qualitative research is needed to better understand the scale of alcohol use in the current employment context, how it is embedded in social norms related to particular industries and the reproduction of the masculine 'worker' identity.

Beliefs that alcoholism is untreatable and alcoholics are beyond help meant that those suffering with alcohol problems, and their close relatives, sometimes do not try to seek treatment. In the descriptions of drinking careers, there was a sharp contrast between 'normal' everyday drinking, which was often tolerated, approved of and might include occasional *zapois*, and 'heavy drinking' when the man lost control, and was labelled an 'alcoholic'. 'Alcoholics' were strongly disapproved of and stigmatised, and alcohol abuse was seen as a social, rather than health problem. There was little perception of a stage in the man's career when treatment might have been effective. More work could be done to build public awareness of how alcohol problems develop and what can be done to treat them. In addition, some of the accounts complained that state-run services were inadequate. One relative stated that a narcology clinic refused to provide treatment as the man was a repeat patient, and others complained about poor organisation and lack of referrals. Some of the respondents were sceptical about the effectiveness of 'coding' treatment.

It was striking that virtually all of the respondents described the men as taking a passive, helpless stance to alcohol, alcohol treatment, and general health. They often refused to go the doctors, even when seriously ill, take prescribed medication, take advice from narcologists, or attempt to reduce their alcohol intake by themselves. Accounts of passivity could be a technique of remembrance for the relative, helping them to explain the death and assign responsibility. Reluctance to seek help could also reflect dissatisfaction and disillusionment with available treatment options for alcoholism. But the findings are in line with other studies arguing that post-Soviet Russian culture does not give a central place to people taking personal responsibility for their own health. Instead there is a persistence of an outlook fostered in the Soviet period which emphasised paternal protection and authority of medical personnel[17,37]. Other scholars point out how more generally, state socialism fostered a sense that events were beyond individual's control [24]. A passive approach, or a rejection of medical authority, was also perceived as an expected masculine characteristic, which his wife could try, but often failed, to influence.

Explanations for excessive drinking and death varied according to the respondent's structural and emotional relationship to the deceased. Children, siblings, and divorced widows more readily attributed personal blame to the man for losing employment, drinking heavily and refusing treatment, whereas those still married emphasised his victim status and his helplessness in the face of difficult external conditions. This contrasted with more uniform 'lay' explanations found elsewhere[27].

There are some methodological limitations to the study. The purposive sampling technique, in which we selected men whose proxy informant believed that alcohol contributed to the man's death, means that the accounts are likely to depict alcohol as strongly related to employment, health and family relations. For this reason the study cannot throw light on the more general association of alcohol with social factors, as would be seen in a more general sample. The biography approach and order of topics may have encouraged the respondent to construct explanations which are logical, ordered and coherent [38]. The use of contemporaneous note taking rather than recording could have introduced both observer bias and recall bias on behalf of the interviewers. The interviewer's own recollection of the interview forces a structure onto the biography which is not necessarily the interviewee's own, and prohibits deeper analysis. After the analysis stage, we would have gained feedback on our findings to increase the validity of our interpretations.

## Conclusions

This study highlights how, among men that died prematurely of alcohol-related causes, negative life events and heavy drinking are mutually reinforcing, producing a sharp decline in health status. Changes in the workplace connected to post-soviet transformation were frequently cited as reasons for starting heavy drinking. Importantly, however, the accounts revealed that industrial, factory, and manual workplace contexts encouraged, or at least tolerated, a culture of heavy and hazardous drinking that probably extend back well into the Soviet period. These practices may well interact with both socio-economic status and ideas about masculinity, and deserves further exploration. Rather than the 'lay' or generalised perceptions about drinking found in previous qualitative work, this study describes how hazardous drinking was personally tolerated, negotiated, and accepted within couples and families in Russia. The broad range of topics covered in the interviews - hazardous drinking patterns, surrogates, attitudes towards treatment can assist in planning interventions that are salient in the Russian context, and hence most likely to be effective.

## Competing interests

The authors declare that they have no competing interests.

## Authors' contributions

LS designed the study, collected and analysed the data; KK did a secondary analysis of the data and wrote the manuscript; NB helped to draft the manuscript; DL helped design the study and draft the manuscript; DE advised on the analysis and helped draft the manuscript. All authors read and approved the final manuscript.

## Pre-publication history

The pre-publication history for this paper can be accessed here:

http://www.biomedcentral.com/1471-2458/11/481/prepub

## References

[B1] Shkolnikov V Andreev E Leon D McKee M Meslé F Vallin J Mortality reversal in Russia: the story so far Hygiea Internationalis 2004 4 4 29 80

[B2] United Nations Statistics Division http://unstats.un.org/unsd/default.htm accessed 30/12/2010 http://unstats.un.org/unsd/default.htm

[B3] Nemtsov AV Alcohol-related human losses in Russia in the 1980s and 1990s Addiction 2002 97 11 1413 1425 10.1046/j.1360-0443.2002.00262.x 12410782

[B4] Leon DA Shkolnikov VM McKee M Kiryanov N Andreev E Alcohol increases circulatory disease mortality in Russia: acute and chronic effects or misattribution of cause? International Journal of Epidemiology 2010 39 5 1279 1290 10.1093/ije/dyq102 20591986 PMC2972439

[B5] Leon DA Chenet L Shkolnikov VM Zakharov S Shapiro J Rakhamnova G Vassin S McKee M Huge variation in Russian mortality rates 1984-94: artefact, alcohol, or what? Lancet 1997 350 9075 383 388 10.1016/S0140-6736(97)03360-6 9259651

[B6] Leon D Saburova L Tomkins S Andreev E Kiryanov N McKee M Shkolnikov V Hazardous alcohol drinking and premature mortality in Russia: a population based case-control study The Lancet 2007 369 9578 2001 2009 10.1016/S0140-6736(07)60941-6 17574092

[B7] Zaridze D Brennan P Boreham J Boroda A Karpov R Lazarev A Konobeevskaya I Igitov V Terechova T Boffetta P Peto R Alcohol and cause-specific mortality in Russia: a retrospective case-control study of 48557 adult deaths The Lancet 2009 373 9682 2201 2214 10.1016/S0140-6736(09)61034-5 PMC2715218 19560602

[B8] Chenet L McKee M Leon D Shkolnikov V Vassin S Alcohol and cardiovascular mortality in Moscow; new evidence of a causal association Journal of Epidemiology and Community Health 1998 52 12 772 10.1136/jech.52.12.772 10396517 PMC1756648

[B9] Pridemore W Heavy Drinking and Suicide in Russia Soc Forces 2006 85 1 413 430 10.1353/sof.2006.0138 17160138 PMC1642767

[B10] WHO WHO Global Status Report on Alcohol 2004 2004 Geneva: World Health Organisation

[B11] Tomkins S Saburova L Kiryanov N Andreev E McKee M Sckolnikov V Leon D Prevalence and socio-economic distribution of hazardous patterns of alcohol drinking: study of alcohol consumption in men aged 25-54 years in Izhevsk, Russia Addiction 2007 2007 102 4 10.1111/j.1360-0443.2006.01693.x PMC1890567 17362291

[B12] Wechsler H Nelson TF Relationship Between Level of Consumption and Harms in Assessing Drink Cut-Points for Alcohol Research: Commentary on "Many College Freshmen Drink at Levels Far Beyond the Binge Threshold" by White et al Alcoholism: Clinical and Experimental Research 2006 30 6 922 927 10.1111/j.1530-0277.2006.00124.x 16737449

[B13] Bobrova N West R Malutina D Koshkina E Terkulov R Bobak M Drinking alcohol surrogates among clients of an alcohol-misuser treatment clinic in Novosibirsk, Russia Subst Use Misuse 2009 44 13 1821 1832 10.3109/10826080802490717 20001282 PMC3941122

[B14] McKee M Suzcs S Sarvary A Adany R Kiryanov N Saburova L Tomkins S Andreev E Leon DA The Composition of Surrogate Alcohols Consumed in Russia Alcoholism: Clinical & Experimental Research 2005 29 10 1884 1888 10.1097/01.alc.0000183012.93303.90 16269919

[B15] Gil A Polikina O Koroleva N McKee M Tomkins S Leon D Availability and characteristics of nonbeverage alcohols sold in 17 Russian cities in 2007 Alcoholism: Clinical and Experimental Research 2009 33 1 79 85 10.1111/j.1530-0277.2008.00813.x 19018753

[B16] Fleming PM Drug and Alcohol User Treatment/Intervention Services in Russia-A Western Perspective Substance Use & Misuse 1996 31 1 103 114 10.3109/10826089609045801 8838396

[B17] Raikhel E Post-Soviet Placebos: Epistemology and Authority in Russian Treatments for Alcoholism Culture, Medicine and Psychiatry 2010 34 1 132 168 10.1007/s11013-009-9163-1 19967435

[B18] Elovich R Drucker E On drug treatment and social control: Russian narcology's great leap backwards Harm Reduction Journal 2008 5 23 10.1186/1477-7517-5-23 PMC2474597 18577225

[B19] Zohoori N Mroz TA Popkin B Glinskaya E Lokshin M Mancini D Kozyreva P Kosolapov M Swafford M Monitoring the economic transition in the Russian Federation and its implications for the demographic crisis -- the Russian Longitudinal Monitoring Survey World Development 1998 26 11 1977 1993 10.1016/S0305-750X(98)00099-0

[B20] Gerber T Hout M More Shock than Therapy: Market Transition, Employment and Income in Russia, 1991-1995 The American Journal of Sociology 1998 104 1

[B21] Stuckler D King L McKee M Mass privatisation and the post-communist mortality crisis: a cross-national analysis The Lancet 2009 373 9661 399 407 10.1016/S0140-6736(09)60005-2 19150132

[B22] Ashwin S ed Adapting to Russia's New Labour Markets: Gender and Employment Behaviour 2006 Oxford: Routledge

[B23] Field M Twigg J eds Russia's Torn Safety Nets: Health and Social Welfare during the Transition 2000 New York: St.Martin's Press

[B24] Watson P Explaining rising mortality among men in Eastern Europe Social Science & Medicine 1995 41 7 923 934 10.1016/0277-9536(94)00405-I 8545667

[B25] Ashwin S Lytkina T Men in crisis in Russia: The role of domestic marginalization Gender & Society 2004 18 2 18921698650

[B26] Pridemore WA Tomkins S Eckhardt K Kiryanov N Saburova L A case-control analysis of socio-economic and marital status differentials in alcohol- and non-alcohol-related mortality among working-age Russian males Eur J Public Health 2010 ckq019 10.1093/eurpub/ckq019 PMC2943507 20219866

[B27] Pietilä I Rytkönen M 'Health is not a man's domain': lay accounts of gender difference in life-expectancy in Russia Sociology of Health & Illness 2008 30 7 1070 1085 10.1111/j.1467-9566.2008.01106.x 18564970

[B28] Pietilä I Rytkönen M Coping with stress and by stress: Russian men and women talking about transition, stress and health Social Science & Medicine 2008 66 2 327 338 10.1016/j.socscimed.2007.09.002 17949875

[B29] Bobrova N West R Malyutina D Malyutina S Bobak M Gender Differences in Drinking Practices in Middle Aged and Older Russians Alcohol and Alcoholism 2010 45 6 573 10.1093/alcalc/agq069 21075855 PMC3943390

[B30] Government of the Russian Federation 2010 Moscow

[B31] Soleman N Chandramohan D Shibuya K Verbal autopsy: current practices and challenges Bulletin of the World Health Organization 2006 84 239 245 10.2471/BLT.05.027003 16583084 PMC2627297

[B32] Segal B The Drunken Society: Alcohol Abuse and Alcoholism in the Soviet Union, A Comparitive Study 1990 New York: Hippocrene Books

[B33] Treml VG Alcohol in the USSR: A Statistical Study 1982 Durham, N.C.: Duke Press Policy Studies

[B34] Hands M Dear G Co-dependency: a critical review Drug and Alcohol Review 1994 13 4 437 445 10.1080/09595239400185571 16818359

[B35] Simpura J Paakkanen P Drinking contexts in Moscow 1994 Demystifying Russian Drinking: Comparative Studies from the 1990s 1997 Helsinki: STAKES

[B36] Cockerham WC Health lifestyles in Russia Social Science & Medicine 2000 51 9 1313 1324 10.1016/S0277-9536(00)00094-0 11037219

[B37] Cockerham WC Snead MC DeWaal DF Health Lifestyles in Russia and the Socialist Heritage Journal of Health and Social Behavior 2002 43 1 42 55 10.2307/3090244 11949196

[B38] Jarvinen M The Biographical Illusion: Constructing Meaning in Qualitative Interviews Qualitative Inquiry 2000 6 3 370 391 10.1177/107780040000600306

